# Macronutrient Composition and Sodium Intake of Diet Are Associated with Risk of Metabolic Syndrome and Hypertension in Korean Women

**DOI:** 10.1371/journal.pone.0078088

**Published:** 2013-10-25

**Authors:** Hea Young Oh, Mi Kyung Kim, Myoungsook Lee, Young Ok Kim

**Affiliations:** 1 Translational Epidemiology Branch, Division of Cancer Epidemiology and Prevention, National Cancer Center, Goyang, Korea; 2 Department of Food and Nutrition, Sungshin Women's University, Seoul, Korea; 3 Department of Food and Nutrition, College of Natural Science, Dongduk Women’s University, Seoul, Korea; INRCA, Italy

## Abstract

Hypertension and hypertriglycemia are the most important contributors to metabolic syndrome (MetS) and cardiovascular disease risk in South Koreans with a relatively lean body mass. These major contributors differ from those identified in Western populations. This study aimed to identify the characteristics of the Korean diet associated with increased risk of MetS, whose prevalence has been steadily increasing in South Korea. On the basis of data collected from 5,320 subjects by the 2007–2008 Korean National Health and Nutrition Examination Survey, 3 dietary patterns were identified using factor analysis and their association with the risk of MetS and its components was examined. The balanced Korean diet, a typical Korean diet of rice and kimchi intake supplemented by a variety of foods had a desirable macronutrient composition and was associated with a lower risk of elevated blood pressure (OR=0.61, 95% CI=0.45–0.84) and hypertriglyceridemia (0.69, 0.49–0.88) in men and a lower risk of elevated blood pressure (0.59, 0.41–0.85) and MetS (0.67, 0.47–0.96) in women. The unbalanced Korean diet, characterized by a high intake of carbohydrates and sodium and little variety, was associated with a higher risk of MetS (1.44, 1.03–2.01) and elevated blood pressure (1.41, 1.00–1.98) in women. The semi-western diet, characterized by a relatively high intake of meat, poultry, and alcohol, was associated with a lower risk of low high-density lipoprotein cholesterol (0.70, 0.54–0.89) in women. Thus, macronutrient composition and sodium intake are associated with the risk of MetS and prehypertension in women. Maintaining a desirable macronutrient composition and avoiding excessive consumption of carbohydrates and sodium should be emphasized for prevention of MetS and hypertension in South Korean women.

## Introduction

The incidence of metabolic syndrome (MetS) has been steadily increasing in South Korea. Having increased from 24.9% in 1998 to 32.4% in 2007–2008 [1], the MetS rate is now higher in South Korea than in other Pacific Rim countries, including Japan (22.0% in 2000) [2] and Australia (22.2% in 1999-2000) [3] and is approaching that in the United States (34.6% in 1999–2000) [4]. At the same time, the rate of hypertension and prehypertension has increased in South Korea such that their prevalence is similar to that in Western countries [5]. 

Although South Koreans tend to be relatively lean and consume less fat, fewer calories, and more plant foods than Western populations, prevalence of MetS is similar in South Korea and Western countries. Interestingly, whereas obesity and insulin resistance are regarded as the central risk factors for MetS in Western countries, hypertension [6] and dyslipidemia [1] are believed to be the most important risk factors for MetS in South Korea. As diet is undoubtedly one of the most important contributors to the development of MetS, it could be a key factor in these international differences. Recently, several studies that analyzed data collected from men and women aged 19 years and above by the 2005 Korean National Health and Nutrition Examination Survey (KNHANES) [7,8] or data from women residing in specific regions [9] reported an association between the risk of MetS and the major dietary patterns of the South Korean population. 

The present study analyzed publicly available data (http://knhanes.cdc.go.kr/knhanes/) collected by the 2007–2008 KNHANES to investigate the association between the risk of MetS and its five components and the major dietary patterns of South Korean in men and women aged 30 to 80 years.

## Methods

### Study population

The KNHANES is a series of cross-sectional surveys of nationally representative samples of the civilian Korean population aged 1 year and above that are conducted annually to assess the health and nutrition status of the South Korean population. To obtain representative samples, the KNHANES uses a stratified, multistage, cluster probability sampling design according to geographical area, age, and gender. More details regarding the sampling method are provided elsewhere [10]. The main components of the overall KNHANES survey are a health interview, health examination survey and nutrition survey. The mean national response rate to the health examination survey was 65.8% (ranging from 53.2% in Daejeon to 86.2% in Jeju) in 2007 and 74.3% (ranging from 63.3% in Busan to 84.9% in Chungbuk) in 2008. 

Data collected by the 2007-2008 KNHANES were analyzed in the present study. Of the 14,338 subjects, 5,320 (2,239 men and 3,081 women) who met the eligibility criteria were included as participants. The inclusion criteria were (1) availability of 24-hour dietary recall and health examination data and age between 30 and 80 years at the time of the survey. The exclusion criteria were (1) following of a dietary regimen accounting for ≤500 or ≥5,000 kcal/day (2), pregnancy and/or lactation for women (3), cancer at the time of the survey.

### Ethics statement

The KNHANES has been performed since 1998 subsequent to receiving ethical approval by Institutional Review Board of Korea Center for Disease Control & Prevention. All subjects provide informed consent prior to participation in this survey.

### Data collection and diagnostic criteria

Trained interviewers had conducted the health interview and health behavior surveys in the subjects’ households to collect demographic and socioeconomic data. The health examination data collected had included waist circumference (WC); height; weight; and levels of high-density lipoprotein cholesterol (HDLc), triglycerides (TG), blood pressure (BP), and fasting blood glucose (FBG). Trained examiners had measured body weight to 0.1 of a kg using a calibrated balance-beam scale, body height to 0.1 of a cm in the upright position using a stadiometer, and WC to 0.1 of a cm at the midpoint between the bottom of the rib case and the top of the lateral border of the iliac crest during minimal respiration. The data were subsequently used to determine body mass index (BMI), calculated by dividing weight in kilograms by the square of the height in meters. BP had been measured 3 times in succession after a 5-min rest period using a mercury sphygmomanometer on the right arm while in a sitting position for determination of the average value of the 3 measurements, which was reported as the final systolic BP (SBP) and diastolic BP (DBP). MetS was diagnosed based upon Adult Treatment Panel-III of the National Cholesterol Education Program (NCEP-ATPIII) criteria with the exception of WC, which was based on the guidelines for Asian populations developed by the International Diabetes Federation [11]. Subjects were classified as having MetS if they had any 3 or more of the following; (1) WC: >90cm (men) and >80cm (women) (2) HDLc: <40mg/dL (men) and <50mg/dL (women), (3) TG: ≥150mg/dL, (4) SBP/DBP: ≥130/85 mm/Hg, 5) FBG: ≥110 mg/dL.

### Assessment of diet and identification of dietary patterns

Trained dietitians explained the method used to complete the 24-hour dietary recall and the means of collecting detailed descriptions of the types and amount of all foods within the 33 food groups listed in Table S1 to the subjects. Total intake of energy and of 17 macronutrients and micronutrients, including energy, protein, fat, carbohydrate, fiber, calcium, sodium, vitamin A, and vitamin C was calculated using the Diet Analysis Plus program. Nutrient intake was calculated based on the Standard Tables of Food Composition of Korea [12], and the classification of food groups was based on the National Nutrition Survey of Korea [13]. Using the FACTOR procedure in SAS V9.2 (SAS Institute, Cary, NC, USA), principal component analysis (PCA) was performed to separately identify the dietary patterns of the male and female subjects. Orthogonal transformation (varimax rotation) of the factors was performed and the number of factors was determined based on eigenvalues greater than 1.5, Scree plot test and the interpretability of the factors. We categorized the quintile cut-offs of dietary pattern scores based on the factor scores of the controls. The factor scores for each subject were used to examine the characteristics of subjects who followed each dietary pattern and determine the associations between the dietary patterns and risk of MetS. 

### Statistical analysis

Analytical processing was conducted using the SURVEY procedures provided in the SAS software. Distributions of general characteristics are presented as mean ± standard deviation (SD) values and frequencies. Dietary pattern scores were divided into quintiles (Q1-Q5), and the means and frequencies were calculated for comparison of the adjusted mean ± standard error (SE). The mean ± SE values for nutrient intake, continuous sociodemographic variables, and biochemical factors and the linear trends across the quintiles of dietary pattern scores were calculated using PROC GLM. Chi-square analysis was performed to investigate the associations between dietary patterns and categorical variables. Multivariate logistic regression analysis was performed to assess the risk of MetS and calculate the odds ratio (OR) and 95% confidence intervals (CI) for each quintile compared with the lowest quintile (Q1). The P values used to test for linear trends were calculated using dietary pattern scores as continuous variables after adjustment for confounding factors.

## Results

### Characteristics of the 3 major dietary patterns

Three dietary patterns were identified by factor analysis (Table S2). The first is the balanced Korean diet, a typical Korean diet of rice and kimchi supplemented by a variety of other food items, including whole grains, fish, sea products, vegetables, fruits, dairy products, eggs, meats, and mushrooms. The macronutrient composition (carbohydrate:protein:fat ratio) of the diets of subjects in the highest quintile (Q5) of the balanced Korean diet, was 66.9:14.5:18.5 for men and 65.5:15.5:19.0 for women (Table 1), within the Korean Nutrition Society-recommended ratio ranges of 55-70:7-20:15-25. In contrast, the macronutrient composition of the unbalanced Korean diet, characterized by little variety and based almost solely on rice and kimchi intake and excessive carbohydrate intake, was not within the recommended ratio ranges (76.6:12.9:10.5 for women and 73.2:14.0:12.8 for men in the Q5). The semi-western diet, characterized by a relatively high intake of meats, poultry, eggs, vegetables, and alcoholic beverages, has a macronutrient composition that falls within the recommended ranges. However, among the subjects in Q5 of all 3 dietary patterns, those in Q5 of the semi-western diet had the highest fat and lowest carbohydrate intake, approaching the maximum and minimum recommendation limits, respectively. 

**Table 1:** Dietary compositions by the quintiles of major three Korean diets based on the KNANES 2007-2008 (n=5,320, 2,239 men and 3,081 women).

		Balanced Korean diet				Unbalanced Korean diet				Semi-Western diet			
Dietary components		Q1	Q3	Q5	*P^c^*	Q1	Q3	Q5	*P*	Q1	Q3	Q5	*P*
Energy (kcal, ×10^3^)	M	2.1 ± 0.03	1.9 ± 0.03	2.4 ± 0.03	**	2.1 ± 0.03	2.0 ± 0.03	2.3 ± 0.03	**	1.7 ± 0.03	2.0 ± 0.03	2.7 ± 0.03	**
	W	1.2 ± 0.02	1.5 ± 0.02	2.0 ± 0.02	**	1.6 ± 0.02	1.5 ± 0.02	1.7 ± 0.02	**	1.7 ± 0.02	1.5 ± 0.02	1.6 ± 0.02	**
Carbohydrate (%)	M	63.1 ^a^	70.1	66.9	**	61.0	69.8	73.2	**	75.0	70.5	59.5	**
	W	77.1	73.1	65.5	**	66.9	71.4	76.6	**	72.9	74.6	63.3	**
Protein (%)	M	17.6	14.5	14.5	0.08	16.6	14.6	14.0	0.12	12.6	14.8	17.5	**
	W	11.9	13.2	15.5	**	13.9	13.8	12.9	**	13.5	13.0	15.4	**
Fat (%)	M	19.3	15.3	18.5	**	22.4	15.6	12.8	**	12.4	14.8	23.0	**
	W	11.0	13.7	19.0	**	19.2	14.8	10.5	**	13.5	12.5	21.3	**
Fiber (g)	M	7.3 ± 0.2	8.1 ± 0.2	9.1 ± 0.2	**	6.4 ± 0.2	8.0 ± 0.2	10.2 ± 0.2	**	7.7 ± 0.2	8.1 ± 0.2	8.7 ± 0.2	*
	W	5.6 ± 0.2	6.7 ± 0.2	8.5 ± 0.2	**	6.0 ± 0.2	6.9 ± 0.2	7.7 ± 0.2	**	8.9 ± 0.2	6.8 ± 0.2	5.4 ± 0.2	**
Calcium (g)	M	0.44 ± 0.01	0.51 ± 0.01	0.65 ± 0.01	**	0.47 ± 0.01	0.50 ± 0.01	0.59 ± 0.01	**	0.46 ± 0.01	0.54 ± 0.01	0.61 ± 0.02	**
	W	0.32 ± 0.01	0.40 ± 0.01	0.54 ± 0.01	**	0.42 ± 0.01	0.40 ± 0.01	0.41 ± 0.01	0.64	0.51 ± 0.01	0.42 ± 0.01	0.33 ± 0.01	**
Sodium (g)	M	5.9 ± 0.1	5.5 ± 0.1	5.4 ± 0.1	*	4.7 ± 0.1	5.4 ± 0.1	7.1 ± 0.1	**	5.3 ± 0.1	5.3 ± 0.1	6.5 ± 0.1	**
	W	3.7 ± 0.1	3.8 ± 0.1	4.7 ± 0.1	**	3.2 ± 0.1	3.9 ± 0.1	4.9 ± 0.1	**	4.3 ± 0.1	3.9 ± 0.1	4.1 ± 0.1	*
Vitamin A (μg, RE ^b^)	M	731 ± 43	771 ± 42	1070 ± 44	**	629 ± 43	806 ± 42	1006 ± 43	**	659 ± 45	773 ± 42	1128 ± 49	**
	W	468 ± 30	633 ± 27	934 ± 31	**	604 ± 28	664 ± 27	706 ± 28	0.07	751 ± 28	662 ± 27	610 ± 27	*
Vitamin C (mg)	M	84.2 ± 3.5	100.5 ± 3.5	139.2 ± 3.6	**	78.7 ± 3.6	99.9 ± 3.5	131.9 ± 3.5	**	90.6 ± 3.7	99.4 ± 3.5	127.7 ± 4.0	**
	W	69.2 ±3.3	94.7 ± 2.9	129.2 ± 3.4	**	96.6 ± 3.0	95.6 ± 2.9	95.5 ± 3.0	0.10	134.2 ± 3.1	90.6 ± 3.0	70.4 ± 3.0	**

Using factor analysis, three major Korean dietary patterns were identified; 1) the balanced Korean diet, characterized by variety and desirable composition of energy-yielding macronutrients, 2) the unbalanced Korean diet, characterized by excessive carbohydrate and sodium intake with monotonous foods, and 3) the semi-Western diet, characterized by relatively high meat, poultry, eggs and alcohol consumption.

The factors were standardized continuous variables, and each subject had a score for each factor.

Daily intake of dietary components for men (M) and women (W) with 30~80 years of age is displayed separately and expressed as mean ± standard error.

Q1, Q3 and Q5 are the lowest, middle and highest quintiles, respectively. ^a^ The value is calculated by the percentage of energy obtained from carbohydrate, protein and fat, respectively and presented as means of the values.

b RE means a retinol equivalent. One RE corresponds to 1μg retinol, 6μg β-carotene in food and 12μg of the other carotenes.

c *P* value to test for linear trend.

** *P* for trend <0.001; *, *P* for trend < 0.05

With regard to sodium consumption, intake was the lowest in men in Q5 of the balanced Korean diet (5.4 g/day) and significantly decreased by quintile. In contrast, intake was the highest in men (7.1 g/day) and women (4.9 g/day) in Q5 of the unbalanced Korean diet and significantly increased by quintile. With regard to other nutrients, men and women with a higher balanced Korean dietary pattern score tended to have higher intakes of fiber, calcium, vitamin A, and vitamin C. In contrast, intake of calcium, vitamin A, and vitamin C did not differ between quintiles in women consuming an unbalanced Korean diet, but tended to decrease by quintile in women consuming a semi-western diet.

### Lifestyle, socioeconomic, and biochemical characteristics of subjects following the 3 major dietary patterns

Subjects of both sexes who scored high for the balanced Korean diet tended to be younger, be more educated, have a higher income level, to smoke less, and likely to drink less drink alcohol (Table 2). In contrast, subjects of both sexes who scored high for the unbalanced Korean diet tended to be older, be less educated, and have a lower income level. Subjects of both sexes who scored high for the semi-western diet tended to be younger, be more educated, have a higher educational level, to smoke more, and to drink more alcohol. Male and female subjects in Q5 of the balanced Korean diet tended to have a significantly lower BP than those in Q1 (Table 3). 

**Table 2:** General characteristics by the quintiles of major three Korean diets based on the KNANES 2007-2008 (n=5,320, 2,239 men and 3,081 women).

		Balanced Korean diet				Unbalanced Korean diet				Semi-Western diet			
Characteristics		Q1	Q3	Q5	*P* ^b^	Q1	Q3	Q5	*P*	Q1	Q3	Q5	*P*
Subjects (n)	M	447	448	448		447	448	448		447	448	448	
	W	616	617	616		616	617	616		616	617	616	
Age (year)	M	54.3 ± 12.9	54.5 ± 14.0	47.7 ± 13.1	**	47.7 ± 13.2	54.4 ± 13.9	55.6 ± 12.8	**	57.9 ± 14.3	53.4 ± 13.3	47.3 ± 11.6	**
	W	59.6 ± 14.1	51.0 ± 13.4	46.8 ± 11.9	**	45.7 ± 11.8	53.1 ± 14.2	57.1 ± 13.6	**	51.3 ± 12.9	54.9 ± 14.3	47.3 ± 13.0	**
MET ^a^	M	73.5 ± 7.0	76.6 ± 7.1	79.3 ± 7.1	0.71	70.0 ± 7.2	71.9 ± 7.0	95.8 ± 7.0	*	66.8 ± 7.1	77.2 ± 7.0	73.5 ± 7.1	0.83
	W	58.0 ± 7.1	63.3 ± 6.9	59.7 ± 7.0	0.11	56.0 ± 7.0	54.4 ± 6.9	79.3 ± 7.0	*	49.7 ± 6.9	59.1 ± 6.9	68.5 ± 7.0	0.36
Current smoker (%)	M	51.4	45.1	41.1	*	51.7	45.4	39.9	**	40.0	42.1	46.2	**
	W	6.8	7.0	5.7	*	6.2	5.4	4.9	0.52	2.4	4.7	10.1	**
Drink (%): ≥ 1 time/week	M	68.5	38.2	28.4	*	45.6	41.2	40.3	**	37.8	44.4	49.6	**
	W	10.7	7.6	10.4	**	12.8	8.6	6.3	**	5.0	6.7	18.5	**
Income (%)	M	44.5	50.7	67.5	**	64.8	51.2	48.2	**	40.7	51.9	69.5	**
: ≥ Middle school	W	30.0	53.9	62.4	**	63.2	45.1	37.1	**	54.1	44.2	57.3	**
Education (%)	M	50.8	57.7	77.4	**	73.8	57.8	44.1	**	49.2	58.1	74.6	**
: ≥ High school	W	25.6	48.9	67.1	**	70.7	45.5	29.2	**	50.5	40.7	62.6	**
Menopause (%)	W	73.2	48.5	33.9	**	32.2	54.9	68.2	**	51.8	59.2	36.0	**

General characteristics for men (M) and women (W) with 30~80 years of age is displayed separately and values are expressed as mean ± standard deviation or percentage of subjects,

Q1, Q3 and Q5 are the lowest, middle and highest quintiles, respectively.

a Metabolic equivalent task (MET) is a measurement of oxygen uptake in a sitting, resting person, varing with age, sex, race, and other factors and used to determine physical activity of subjects. MET value is expressed as mean ± standard error.

^b^ *P* value to test for linear trend; ** p for trend < 0.001, * p for trend < 0.05

Life style and socioeconomic factors were adjusted for age.

**Table 3:** Biochemical characteristics by the quintiles of major three Korean diets based on the KNANES 2007-2008 (n=5,320, 2,239 men and 3,081 women).

		Balanced Korean diet				Unbalanced Korean diet				Semi-Western diet			
Dietary components		Q1	Q3	Q5	*P* ^a^	Q1	Q3	Q5	*P*	Q1	Q3	Q5	*P*
Body mass index (kg/m^2^)	M	23.8 ± 0.1	23.6 ± 0.1	23.8 ± 0.1	*	23.7 ± 0.1	23.8 ± 0.1	23.8 ± 0.1	0.97	23.5 ± 0.1	23.5 ± 0.1	24.0 ± 0.1	*
	W	23.3 ± 0.1	23.5 ± 0.1	23.4 ± 0.1	0.45	23.4 ± 0.1	23.3 ± 0.1	23.4 ± 0.1	0.76	23.5 ± 0.1	23.4 ± 0.1	23.4 ± 0.1	0.84
Waist circumference (cm)	M	85.2 ± 0.4	84.0 ± 0.4	84.4 ± 0.4	*	84.8 ± 0.4	84.6 ± 0.4	84.9 ± 0.4	0.82	84.0 ± 0.4	83.6 ± 0.4	85.8 ± 0.4	**
	W	79.4 ± 0.4	80.4 ± 0.4	80.2 ± 0.4	0.34	79.9 ± 0.4	79.7 ± 0.4	80.2 ± 0.4	0.82	80.3 ± 0.4	79.7 ± 0.4	80.0 ± 0.4	0.66
Systolic blood pressure	M	123 ± 0.7	121 ± 0.7	119 ± 0.7	**	122 ± 0.7	120 ± 0.7	122 ± 0.7	0.18	120 ± 0.7	121 ± 0.7	122 ± 0.7	0.42
(mmHg)	W	118 ± 0.7	117 ± 0.6	114 ± 0.7	**	114 ± 0.7	116 ± 0.6	117 ± 0.7	0.06	115 ± 0.6	116 ± 0.7	115 ± 0.7	*
Diastolic blood pressure	M	79.4 ± 0.5	78.5 ± 0.5	77.0 ± 0.5	**	79.0 ± 0.5	78.3 ± 0.5	78.3 ± 0.5	0.06	78.2 ± 0.5	78.3 ± 0.5	79.3 ± 0.5	0.31
(mmHg)	W	74.1 ± 0.4	74.6 ± 0.4	72.6 ± 0.4	**	73.3 ± 0.4	73.5 ± 0.4	73.0 ± 0.4	0.28	72.6 ± 0.4	73.9 ± 0.4	73.2 ± 0.4	*
Triglycerol (mg/dL)	M	180 ± 5.9	160 ± 5.9	145 ± 6.0	**	167 ± 6.0	153 ± 5.9	149 ± 5.9	0.13	162 ± 6.0	158 ± 5.9	160 ± 6.0	0.93
	W	127 ± 3.3	118 ± 3.2	117 ± 3.2	0.32	117 ± 3.3	123 ± 3.2	119 ± 3.2	0.35	119 ± 3.2	121 ± 3.2	121 ± 3.2	0.91
HDL cholesterol (mg/dL)	M	47.3 ± 0.6	45.2 ± 0.6	44.7 ± 0.6	**	45.4 ± 0.6	45.1 ± 0.6	46.1 ± 0.6	0.80	44.6 ± 0.6	45.1 ± 0.6	45.9 ± 0.6	0.23
	W	49.3 ± 0.5	49.5 ± 0.5	50.3 ± 0.5	0.43	50.4 ± 0.5	49.0 ± 0.5	49.3 ± 0.5	**	49.1 ± 0.5	49.9 ± 0.5	51.4 ± 0.5	*
Blood glucose (mg/dL)	M	101.0 ± 1.1	98.6 ± 1.1	98.3 ± 1.1	0.28	98.7 ± 1.1	100.0 ± 1.1	98.1 ± 1.1	0.28	99.8 ± 1.1	98.7 ± 1.1	98.7 ± 1.1	0.60
	W	96.5 ± 0.9	97.1 ± 0.9	96.7 ± 0.9	0.90	95.6 ± 0.9	96.4 ± 0.9	97.1 ± 0.9	0.16	97.3 ± 0.9	96.1 ± 0.9	96.6 ± 0.9	0.41

Biochemical characteristics including BMI for men (M) and women (W) with 30~80 years of age are displayed separately, and values are expressed as mean ± standard deviation. Q1, Q3 and Q5 are the lowest, middle and highest quintiles, respectively.

^a^ *P* Value to Test for Linear Trend; **, p for Trend < 0.01, *, p for Trend < 0.05

Biochemical data were adjusted for age, smoking habit, alcohol consumption behavior, physical activity, residence and household income.

### Associations between the dietary patterns and MetS and its 5 components

**Figure 1:**
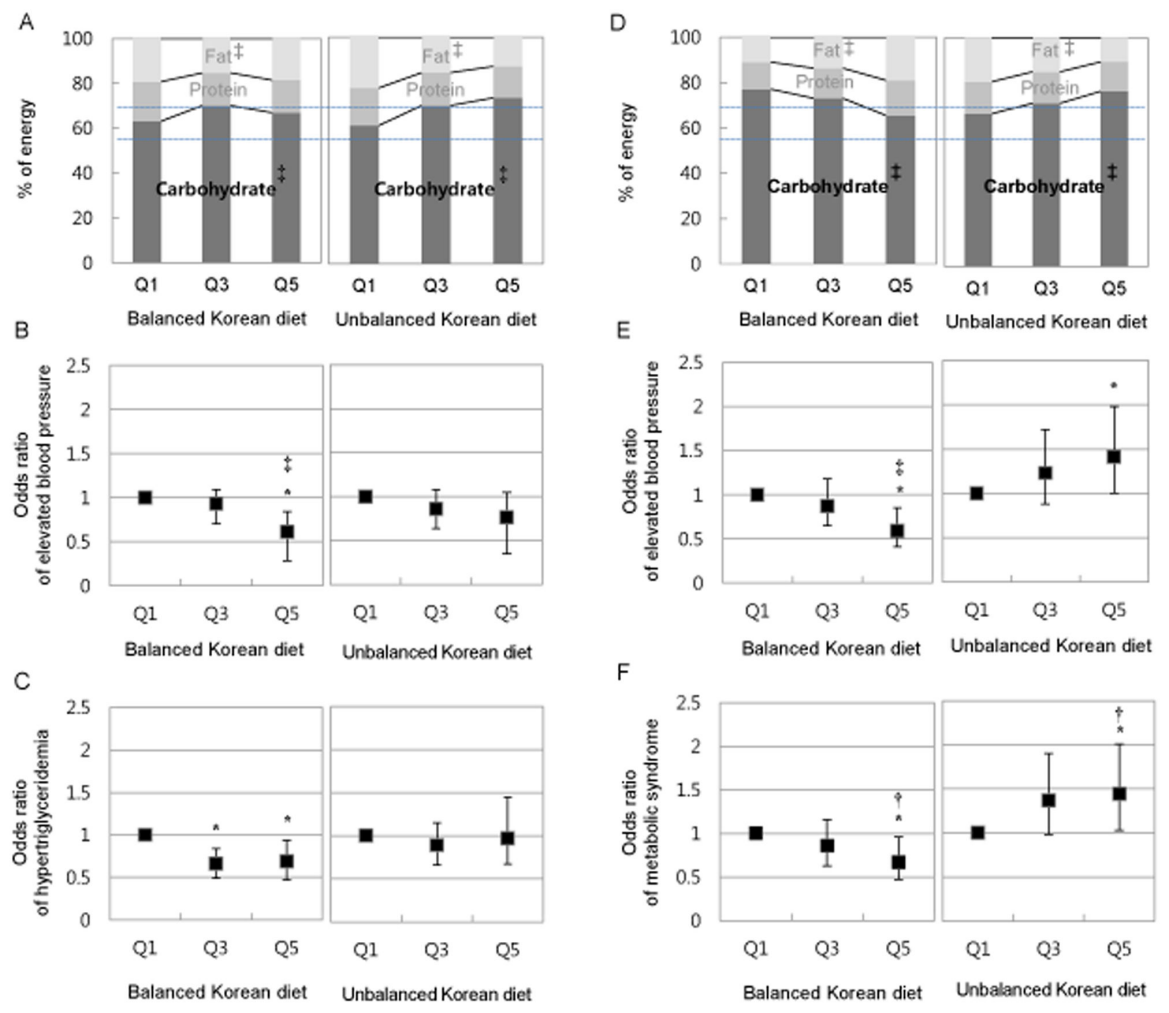
Metabolic syndrome risk and macronutrient composition of balanced Korean diet and unbalanced Korean diet. A and D: Macronutrient composition by quintiles (Q1: lowest, Q3: middle, Q5: highest; Q3 and Q5 was calculated on the base of Q1); % of energy is the percentage of energy obtained from carbohydrate, protein and fat; A dotted line is recommended intake of carbohydrate for Korean; ‡, p for trend <0.001. B and E: Multivariate OR and 95% CI (bar) of elevated blood pressure; *, Significant odd ratio, ‡, p for trend <0.001; Adjusted for age, smoking, alcohol and physical activity. C and F. Multivariate OR and 95% CI (bar) of hypertriglyceridemia for men and metabolic syndrome for women; *, Significant odd ratio, †, p for trend < 0.05; Adjusted for age, smoking, alcohol and physical activity.

The balanced Korean diet was significantly associated with a lower risk of MetS (Table 4). Women in Q5 of this pattern had a multivariate OR for the risk of MetS of 0.67 (95% CI, 0.47~0.96, p for trend < 0.05), which was significantly lower than that of women in Q1. Furthermore, men and women in Q5 of this pattern were associated with a markedly lower risk of elevated BP (0.61, 0.45~0.84, p < 0.001 and 0.59, 0.41~0.85, p < 0.001, respectively). Men in Q5 and Q3 of this pattern were associated with a lower risk of hypertriglyceridemia (0.66, 0.49~0.88 and 0.69, 0.51~0.94, respectively, p = 0.05). Women in Q5 of the unbalanced Korean diet were associated with a significantly higher risk of MetS (1.44, 1.03~2.01, p < 0.05) and elevated BP (1.41, 1.00~1.98, p = 0.17). Women in the Q5 of the semi-western diet were associated with a lower risk of low HDLc (0.70, 0.54~0.89, p < 0.01). Taken together, carbohydrate intake of women following the balanced Korean diet and the unbalanced Korean diet showed opposite trends by quintile (Figure 1D), and the trends of carbohydrate intake of these two diets were associated with that of the risk of elevated blood pressure (Figure 1E) and MetS by quintile (Figure 1F).

**Table 4:** Multivariate adjusted odds ratios and 95% confidence interval for metabolic syndrome and its five components based on the KNHANES 2007-2008 (n=5,320, 2,239 men and 3,081 women).

Metabolic syndrome and		Balanced Korean diet				Unbalanced Korean diet					Semi-western diet		
its components		Q1	Q3	Q5	*P*	Q1	Q3	Q5	*P*	Q1	Q3	Q5	*P*
Metabolic syndrome	M	1	0.85 (0.60-1.20)	0.88 (0.61-1.26)	0.92	1	1.08 (0.76-1.53)	0.99 (0.68-1.45)	0.89	1	0.77 (0.54-1.08)	0.95 (0.66-1.39)	0.64
	W	1	0.86 (0.63-1.16)	0.67 (0.47-0.96)	*	1	1.37 (0.98-1.90)	1.44 (1.03-2.01)	*	1	0.76 ( 0.55-1.04)	0.87 (0.63-1.20)	0.17
Abdominal obesity	M	1	0.69 (0.50-0.96)	0.86 (0.62-1.19)	0.97	1	0.98 (0.71-1.35)	1.02 (0.73-1.44)	0.79	1	0.86 (0.62-1.21)	1.24 (0.88-1.75)	0.15
	W	1	1.12 (0.85-1.47)	1.05 (0.78-1.43)	0.45	1	1.01 (0.77-1.31)	0.96 (0.72-1.27)	0.56	1	0.89 (0.68-1.16)	0.98 (0.75-1.28)	0.73
Hypertriglycemia	M	1	0.66 (0.49-0.88)	0.69 (0.51-0.94)	0.05	1	0.89 (0.66-1.19)	0.97 (0.71-1.32)	0.50	1	0.87 (0.65-1.17)	1.00 (0.73-1.37)	0.84
	W	1	1.05 (0.78-1.41)	0.84 (0.60-1.19)	0.62	1	1.12 (0.83-1.51)	1.06 (0.77-1.45)	0.50	1	0.86 (0.64-1.16)	0.96 (0.70-1.30)	0.67
Low HDL-cholesterol	M	1	1.02 (0.75-1.39)	1.22 (0.89-1.67)	0.78	1	0.92 (0.68-1.24)	0.91 (0.66-1.26)	0.72	1	0.95 (0.70-1.28)	1.05 (0.76-1.46)	0.78
	W	1	0.87 (0.67-1.12)	0.79 (0.59-1.05)	0.21	1	1.27 (0.99-1.63)	1.10 (0.85-1.43)	*	1	0.81 (0.63-1.04)	0.70 (0.54-0.89)	**
Elevated blood pressure	M	1	0.93 (0.70-1.26)	0.61 (0.45-0.84)	***	1	0.86 (0.64-1.17)	0.76 (0.54-1.05)	0.06	1	1.04 (0.77-1.41)	1.12 (0.81-1.56)	0.61
	W	1	0.87 (0.65-1.18)	0.59 (0.41-0.85)	***	1	1.23 (0.88-1.72)	1.41 (1.00-1.98)	0.17	1	1.14 (0.83-1.57)	1.23 (0.88-1.72)	0.51
Abnormal glucose	M	1	0.77 (0.43-1.37)	0.76 (0.40-1.43)	0.50	1	1.28 (0.71-2.30)	1.03 (0.53-2.00)	0.50	1	0.75 (0.44-1.29)	0.63 (0.33-1.22)	0.26
homeostasis	W	1	0.83 (0.47-1.40)	0.81 (0.41-1.59)	0.62	1	2.09 (1.07-4.09)	1.75 (0.87-3.50)	0.12	1	0.78 (0.43-1.39)	0.62 (0.32-1.18)	0.07

Values are expressed as odd ratios and 95% confidence intervals.

Q1, Q3 and Q5 are the lowest, middle and highest quintiles, respectively; Q3 and Q5 were calculated on the base of Q1.

P values is to test for a linear trend of Q1 ~ Q5 for metabolic syndrome and its five components in the regression model. Significances were presented with * (p for trend < 0.05), ** (p for trend < 0.01), and *** (p for trend < 0.001).

Adjusted for age, smoking history, alcohol behavior and physical activity.

Metabolic syndrome was based upon Adult Treatment Panel-III of the National Cholesterol Education Program criteria with the exception of the criterion regarding waist circumference, which was based on the guidelines for Asian populations developed by the International Diabetes Federation.

## Discussion

Many dietary patterns, such as the prudent diet, the hypertension DASH diet, and the Mediterranean diet, have been reported to reduce the risk of stroke, hypertension, MetS, and cardiovascular disease (CVD) [14,15]. These dietary patterns are characterized by a high intake of fruits, vegetables, whole grains, legumes, low-fat dairy products and fish and low intake of red meat, sweets, and refined carbohydrates. The balanced Korean diet, characterized by a nutrient intake similar to that of previously identified healthy dietary patterns was found to be associated with a lower risk of prehypertension and hypertriglyceridemia in men and a lower risk of prehypertension and MetS in women. In contrast, the unbalanced Korean diet, characterized by little variety, high carbohydrate intake, and undesirable macronutrient composition, was associated with increased risk of prehypertension and MetS in women. 

Actually, compared to mean carbohydrate intake reported in the 2007 KNHENS (66.9%) [16], subjects following the unbalanced Korean diet obtained a much higher carbohydrates intake (73.2% and 76.6% for men and women in the Q5, respectively) [8]. Another critical finding was a high consumption of sodium by subjects following the unbalanced Korean diet. Although the average South Korean has a relatively high sodium intake, exceeding the daily maximum levels recommended by the American Health Association (1.5 g/day) and World Health Organization (2 g/day) for all study subjects, that of subjects in Q5 of the unbalanced Korean diet was extremely high (7.1 g/day for men and 4.9 g/day for women).

The adoption of Western dietary patterns in Asian countries has been reported to be an important factor in the increased prevalence of obesity and chronic disease in adolescent men in South Korea [8] and in the Japanese [17] and Chinese [18] populations as a whole. However, previous epidemiological findings have been not consistent. For example, the Japanese traditional dietary pattern, characterized by a high intake of rice, miso soup, and soy products, has been independently associated with a higher risk of obesity in young women [19], and high-carbohydrate consumption has been reported to cause hyperinsulinemia, postprandial hyperglycemia, and hypertriglyceridemia in South Asian adults [20]. Furthermore, several epidemiological studies have reported that low fat and high carbohydrate consumption is associated with CVD [21,22] and that high-saturated fat and sugar intake is associated with accelerated development of cardiac pathology and dysfunction in hypertensive subjects who are not diabetic and only modestly obese [23,24]. In addition, high intake of sodium has been known to be associated with significantly increased risk of stroke, CVD [25], elevated blood pressure, and these links were found in our study subjects and in Korean MetS patients [6]. Taken together, these findings indicate that following a dietary pattern characterized by an undesirable macronutrient composition due to excessive intake of carbohydrates and a high sodium intake may contribute to an elevated risk of MetS as well as cardiac dysfunction in Asian populations despite their relatively lean body mass.

The semi-western diet was not associated with an increased risk of MetS, but was found to be correlated with a lower risk of low HDLc in women. Despite its emphasis on meats, poultry, eggs, and alcohol, this pattern is not identical to the typical Western diet, particularly regarding fat intake. Specifically, fat intakes in the Q5 of this pattern were about 23.0% for men and 21.3% for women, higher than those for subjects in Q5 of the other dietary patterns and approaching the maximum recommended intake, but only approximately 0.7 and 0.6 times those of the average American man and woman, respectively [26]. In addition, men following the semi-western diet had a higher intake of vegetables and moderate intake of fiber, vitamin A, and vitamin B, which might be partially explained by the unique Korean dietary practice of wrapping meats in vegetables. Furthermore, women following this pattern were associated with a lower HDLc, which could explained by a moderate intake of alcohol exerting a beneficial effect on HDLc by increasing the transport rate of HDL apolipoprotein A-I and A-II [27].

In accordance with those of previous studies, the findings of the present study indicate that the association between dietary patterns and the risk of MetS in South Koreans differ according to sex. In one KNHANES study, a high carbohydrate intake (>70% of energy) was significantly associated with the risk of diabetes and low HDLc in women, but inversely associated with total cholesterol in men [28]. In another study comparing the risk of metabolic risk traits among South Koreans according to the rice-eating patterns, the risk of MetS was lower in those who consumed the rice with beans and the rice with multi-grains groups, particularly in postmenopausal women [29]. The dissimilar associations observed in men and women may be attributable to differences in cell metabolism that arise from differences in levels of hormones, which affect insulin effects, blood cholesterol transport and endogenous lipid synthesis [30]. At the same time, food choices [31], food preferences [32], and food behaviors, which may be affected by social desirability and social approval biases [33] appear to vary by gender [34]. 

Although PCA is a subjective process that produces results difficult to replicate in other populations, use of an approach based on analysis of dietary patterns enhances understanding of complex dietary variables compared to use of a single component approach [28]. While acknowledging that prospective research is now required to establish stronger evidence, the findings of this study provide a base on which to further examine the manner in which international differences in dietary intake patterns affect the risk of chronic diseases. 

 In conclusion, the balanced Korean diet, characterized by a desirable macronutrient composition and a variety of food items, was associated with a lower risk of hypertension and MetS, while the unbalanced Korean diet, characterized by an undesirable macronutrient composition due to a high carbohydrate and sodium intake, was associated with a higher risk of MetS, but only in women. In contrast, the balanced Korean diet was not associated with the risk of MetS in men, but was associated with a lower risk of hypertension and hypertriglycerides, while the unbalanced Korean diet was not associated with the risk of MetS in men. These findings indicated that maintaining a desirable macronutrient composition and avoiding excessive consumption of carbohydrates and sodium should be emphasized for prevention of MetS and hypertension in South Korean women. Further studies are needed to clarify the associations between dietary patterns and the risk of MetS in men and identify the factors underlying the differences in the associations for men and women.

## Supporting Information

Table S1 **Thirty three food groupings included in the dietary pattern from food frequency questionnaire of the KNANES 2007-2008.** 1) Foods listed were 24h-recall method.
(DOCX)

Table S2 Identification of dietary pattern from factor-loadings for foods from food frequency questionnaire of the KNANES 2007-2008 (n=5,320, 2,239 men, 3,081 women).
(DOCX)
