# Supplementary material for: Macronutrient Composition and Sodium Intake of Diet Are Associated with Risk of Metabolic Syndrome and Hypertension in Korean Women
Source: PLoS One. 2013 Oct 25;8(10):e78088. doi: 10.1371/journal.pone.0078088 (PMC3808273; doi:10.1371/journal.pone.0078088)
Supplement: Table S1 — Thirty three food groupings included in the dietary pattern from food frequency questionnaire of the KNANES 2007-2008. 1) Foods listed were 24h-recall method. (DOCX) [file pone.0078088.s001.docx]

|  | Food groups | Foods in the group^1)^ |
| --- | --- | --- |
| 1. | Rice | rice, paddy rice, glutenous rice |
| 2. | Whole grain | barley, sorghum, oats, brown rice, buckwheat |
| 3. | Korean style soup | meat soup, vegetable soup, soybean paste soup |
| 4. | Stew | meat, fish, shellfish, vegetables pot stew |
| 5. | Meat | beef, pork, edible viscera, beef product leg, mutton meat |
| 6. | Processed meat | ham, sausage, bacon, dried beef, hamburger patty, beef and pork roll |
| 7. | Poultry | chicken, edible viscera, chicken soup, duck, turkey |
| 8. | Fish | fish with white meat, fish with blue back, fish with red meat |
| 9. | Shellfish | oyster, clam, turban shell, abalone, mussel, crab, squid, shrimp, octopus |
| 10. | Eggs | chicken's egg, quail's egg, duck's egg |
| 11. | Kimchi | all types of kimchi |
| 12. | Vegetables | green and dark yellow vegetables, white vegetables |
| 13. | Mushroom | oyster mushroom, pine mushroom, mushroom, winter fungus |
| 14. | Vegetable oil | perilla oil, margarine, corn oil, olive oil, sesame oil, soybean oil |
| 15. | Nuts | peanut, walnut, almond, pine nut, cashew nut, sunflower seed, acorn |
| 16. | Pickled vegetables | cucumber pickled and preserved with salt, naraseukei |
| 17. | Seaweeds | laver, see tangle, sea mustard, sea lettuce |
| 18. | Pulse | soybean, soybean curd, red bean, soybean milk, green peas, soybean paste |
| 19. | Noodle | instant noodle, Chinese noodle, buckwheat noodle, spaghetti, Udong, dumpling |
| 20. | Bread | loaf bread, sandwich, pizza, pie, hotdog, sponge cake, croissant, hamburger |
| 21. | Soft drink | coke, other carbonated beverages, sports beverage |
| 22. | Alcohol | beer, rice wine, soju, whisky, unstrained rice wine, champagne |
| 23. | Sea products | dried fish, small fish with bone, canned fish, fish egg, fish fermented, fish paste |
| 24. | Dairy products | milk, yogurt, yogurt curd type, cheese, condensed milk, ice milk |
| 25. | Rice cake | all types of rice cake |
| 26. | Fruits | apple, orange, banana, strawberry, grape, peach, pear, kiwi, persimmons, melon, water melon, citrus, plum, apricots, pineapple, canned fruit |
| 27. | Fruit & vegetable juice | vegetable juice, tomato juice, 100% fruit juice, sweetened fruit drinks |
| 28. | Potatoes | white potatoes, french fries, sweet potatoes, corn, taro, starches, |
| 29. | Salt-containing seasonings | soy sauce, soybean paste, red pepper paste, tomato ketchup, mayonnaise, oyster sauce, salty seasonings used during cooking |
| 30. | Sugar | fructose, honey, jam |
| 31. | Confectioneries | crude maltose, jelly, chocolate, caramel, ice cream |
| 32. | Coffee & cocoa | coffee, cocoa |
| 33. | Tea | green tea, black tea, ginseng tea, red ginseng tea, ginger tea, citron tea |
|  | | |

Supplementary table 2. Identification of dietary pattern from factor-loadings for foods from food frequency questionnaire of the KNANES 2007-2008 (n=5,320, 2,239 men, 3,081 women)

|  | Food Groups |  | Men |  |  |  | Women |  |
| --- | --- | --- | --- | --- | --- | --- | --- | --- |
|  |  | Balanced Korean diet | Unbalanced Korean  diet | Semi-Western  diet |  | Balanced Korean diet | Unbalanced Korean  diet | Semi-  Western  diet |
| 1 | Vegetables | 0.17 | 0.10 | 0.61 |  | 0.58 | ― | 0.13 |
| 2 | Vegetable oil | 0.40 | -0.10 | 0.57 |  | 0.60 | ― | 0.31 |
| 3 | Salt-containing seasonings | ― | ― | 0.63 |  | 0.57 | ― | 0.20 |
| 4 | Egg | 0.41 | ― | 0.19 |  | 0.41 | ― | ― |
| 5 | Sugar | 0.13 | ― | 0.39 |  | 0.33 | -0.16 | 0.19 |
| 6 | Mushroom | ― | ― | 0.19 |  | 0.33 | ― | -0.14 |
| 7 | Sea products | 0.41 | ― | 0.17 |  | 0.30 | ― | ― |
| 8 | Fish | ― | ― | 0.23 |  | 0.29 | ― | ― |
| 9 | Shellfish | -0.15 | ― | 0.36 |  | 0.25 | ― | ― |
| 10 | Pulse | ― | -0.13 | ― |  | 0.24 | ― | -0.23 |
| 11 | Seaweeds | ― | 0.15 | ― |  | 0.24 | 0.12 | -0.11 |
| 12 | Nuts | ― | ― | 0.14 |  | 0.18 | ― | -0.16 |
| 13 | Rice | 0.10 | 0.73 | 0.12 |  | 0.13 | 0.73 | -0.12 |
| 14 | Kimchi | -0.11 | 0.50 | ― |  | 0.12 | 0.42 | -0.27 |
| 15 | Korean style soup | 0.27 | 0.30 | ― |  | ― | 0.29 | -0.14 |
| 16 | Tea | ― | -0.13 | 0.11 |  | ― | -0.14 | ― |
| 17 | Noodle | 0.12 | ― | ― |  | ― | -0.15 | ― |
| 18 | Coffee and cocoa | ― | -0.13 | ― |  | ― | -0.20 | 0.11 |
| 19 | Rice cake | 0.27 | -0.10 | -0.11 |  | ― | -0.22 | ― |
| 20 | Soft drink | 0.22 | -0.32 | ― |  | 0.17 | -0.23 | ― |
| 21 | Fruit & vegetable juice | 0.20 | -0.14 | ― |  | ― | -0.31 |  |
| 22 | Confectioneries | 0.34 | -0.11 | ― |  | ― | -0.32 | -0.11 |
| 23 | Bread | 0.25 | -0.28 | -0.13 |  | ― | -0.34 | -0.12 |
| 24 | Dairy products | 0.32 | -0.17 | ― |  | ― | -0.34 | -0.22 |
| 25 | Alcohol | -0.37 | -0.40 | 0.34 |  | ― | -0.16 | 0.42 |
| 26 | Poultry | ― | -0.14 | 0.22 |  | ― | ― | 0.39 |
| 27 | Meat | -0.14 | -0.15 | 0.55 |  | 0.34 | ― | 0.37 |
| 28 | Pickled vegetables | ― | ― | 0.11 |  | ― | ― | 0.28 |
| 29 | Processed meat | 0.26 | -0.14 | ― |  | ― | ― | 0.21 |
| 30 | Potatoes | 0.19 | ― | ― |  | ― | ― | -0.12 |
| 31 | Stew | -0.10 | 0.13 | ― |  | ― | 0.10 | -0.19 |
| 32 | Whole grain | 0.22 | ― | ― |  | ― | -0.16 | -0.32 |
| 33 | Fruits | 0.21 | ― | ― |  | 0.21 | -0.20 | -0.39 |

These data are factor loadings (correlation coefficients between the variables and factors) derived from principal component factor analysis.

Absolute values of factor loadings <0.10 are not listed and indicated by ‘-’ for simplicity.

C
