# Supplementary material for: Macronutrient Composition and Sodium Intake of Diet Are Associated with Risk of Metabolic Syndrome and Hypertension in Korean Women
Source: PLoS One. 2013 Oct 25;8(10):e78088. doi: 10.1371/journal.pone.0078088 (PMC3808273; doi:10.1371/journal.pone.0078088)
Supplement: Table S2 — Identification of dietary pattern from factor-loadings for foods from food frequency questionnaire of the KNANES 2007-2008 (n=5,320, 2,239 men, 3,081 women). (DOCX) [file pone.0078088.s002.docx]

|  |  | Men | | |  | Women | | |
| --- | --- | --- | --- | --- | --- | --- | --- | --- |
|  | Food Groups | Balanced Korean diet | Unbalanced Korean  diet | Semi-Western  diet |  | Balanced Korean diet | Unbalanced Korean  diet | Semi-  Western  diet |
| 1 | Vegetables | 0.17 | 0.10 | 0.61 |  | 0.58 | ― | 0.13 |
| 2 | Vegetable oil | 0.40 | -0.10 | 0.57 |  | 0.60 | ― | 0.31 |
| 3 | Salt-containing | ― | ― | 0.63 |  | 0.57 | ― | 0.20 |
|  | seasonings |  |  |  |  |  |  |  |
| 4 | Egg | 0.41 | ― | 0.19 |  | 0.41 | ― | ― |
| 5 | Sugar | 0.13 | ― | 0.39 |  | 0.33 | -0.16 | 0.19 |
| 6 | Mushroom | ― | ― | 0.19 |  | 0.33 | ― | -0.14 |
| 7 | Sea products | 0.41 | ― | 0.17 |  | 0.30 | ― | ― |
| 8 | Fish | ― | ― | 0.23 |  | 0.29 | ― | ― |
| 9 | Shellfish | -0.15 | ― | 0.36 |  | 0.25 | ― | ― |
| 10 | Pulse | ― | -0.13 | ― |  | 0.24 | ― | -0.23 |
| 11 | Seaweeds | ― | 0.15 | ― |  | 0.24 | 0.12 | -0.11 |
| 12 | Nuts | ― | ― | 0.14 |  | 0.18 | ― | -0.16 |
| 13 | Rice | 0.10 | 0.73 | 0.12 |  | 0.13 | 0.73 | -0.12 |
| 14 | Kimchi | -0.11 | 0.50 | ― |  | 0.12 | 0.42 | -0.27 |
| 15 | Korean style soup | 0.27 | 0.30 | ― |  | ― | 0.29 | -0.14 |
| 16 | Tea | ― | -0.13 | 0.11 |  | ― | -0.14 | ― |
| 17 | Noodle | 0.12 | ― | ― |  | ― | -0.15 | ― |
| 18 | Coffee and cocoa | ― | -0.13 | ― |  | ― | -0.20 | 0.11 |
| 19 | Rice cake | 0.27 | -0.10 | -0.11 |  | ― | -0.22 | ― |
| 20 | Soft drink | 0.22 | -0.32 | ― |  | 0.17 | -0.23 | ― |
| 21 | Fruit & vegetable juice | 0.20 | -0.14 | ― |  | ― | -0.31 |  |
| 22 | Confectioneries | 0.34 | -0.11 | ― |  | ― | -0.32 | -0.11 |
| 23 | Bread | 0.25 | -0.28 | -0.13 |  | ― | -0.34 | -0.12 |
| 24 | Dairy products | 0.32 | -0.17 | ― |  | ― | -0.34 | -0.22 |
| 25 | Alcohol | -0.37 | -0.40 | 0.34 |  | ― | -0.16 | 0.42 |
| 26 | Poultry | ― | -0.14 | 0.22 |  | ― | ― | 0.39 |
| 27 | Meat | -0.14 | -0.15 | 0.55 |  | 0.34 | ― | 0.37 |
| 28 | Pickled vegetables | ― | ― | 0.11 |  | ― | ― | 0.28 |
| 29 | Processed meat | 0.26 | -0.14 | ― |  | ― | ― | 0.21 |
| 30 | Potatoes | 0.19 | ― | ― |  | ― | ― | -0.12 |
| 31 | Stew | -0.10 | 0.13 | ― |  | ― | 0.10 | -0.19 |
| 32 | Whole grain | 0.22 | ― | ― |  | ― | -0.16 | -0.32 |
| 33 | Fruits | 0.21 | ― | ― |  | 0.21 | -0.20 | -0.39 |
